# Elevated Serum Neuropeptide FF Levels Are Associated with Cognitive Decline in Patients with Spinal Cord Injury

**DOI:** 10.1155/2021/4549049

**Published:** 2021-11-11

**Authors:** Shifei Sun, Shilong Sun, Yan Meng, Bin Shi, Yuanzhen Chen

**Affiliations:** ^1^Bone Biomechanics Engineering Laboratory of Shandong Province, Neck-Shoulder and Lumbocrural Pain Hospital of Shandong First Medical University, Jinan 250062, China; ^2^Department of Radiology, China-Japan Friendship Hospital, Beijing 100029, China

## Abstract

**Background:**

Spinal cord injury (SCI) has high incidence globally and is frequently accompanied by subsequent cognitive decline. Accurate early risk-categorization of SCI patients for cognitive decline using biomarkers can enable the timely application of appropriate neuroprotective measures and the development of new agents for the management of SCI-associated cognitive decline. Neuropeptide FF is an endogenous neuropeptide with a multitude of functions and is associated with neuroinflammatory processes. This prospective study investigated the predictive value of serum neuropeptide FF levels measured after acute SCI for subsequent cognitive decline.

**Methods:**

88 patients presenting with acute SCI without preexisting neurological injury, brain trauma, or severe systemic illness and 60 healthy controls were recruited. Serum neuropeptide FF levels, clinical, and routine laboratory variables including low-density lipoprotein, high-density lipoprotein, fasting blood glucose, total triiodothyronine (TT3), total thyroxine (TT4), and thyroid-stimulating hormone (TSH) levels collected from all subjects were assessed. Montreal cognitive assessment (MoCA) was performed 3 months after enrollment. SCI patients were grouped according to quartile of serum neuropeptide FF level and MoCA scores were compared using ANOVA. Additionally, multivariate linear regression with clinical and laboratory variables was performed to predict MoCA scores.

**Results:**

SCI patients displayed significantly higher baseline serum neuropeptide FF levels than healthy controls (38.5 ± 4.1 versus 23.4 ± 2.0 pg/ml, *p* < 0.001^∗∗^). SCI patients in higher quartiles of baseline serum neuropeptide FF displayed significantly lower MoCA scores at 3 months. Linear regression analysis indicated serum neuropeptide FF levels as a significant independent predictor of worse MoCA scores after SCI (*r* = 0.331, *p* = 0.034^∗^).

**Conclusion:**

Early serum neuropeptide FF levels significantly and independently predicted cognitive decline after acute SCI among patients without preexisting neurological disorders.

## 1. Introduction

Spinal cord injury (SCI), involving injury to the vital structures of the spinal column, is highly prevalent worldwide, with a mean prevalence of 1 in 1000, and reported incidence rates from different geographical regions vary from approximately 3 to 195 cases per million per year [1–3]. SCI can occur due to both traumatic and nontraumatic causes, and acute SCI typically occurs due to trauma, with road traffic accidents and falls as the most common causes [1, 3]. Higher incidence of traumatic SCI has been noted in middle- and lower-income countries [3]. Apart from high mortality, SCI can account for high morbidity, disability, healthcare, and economic burden [4–6].

SCI is widely reported to be associated with high rates of cognitive impairment [7–9], and SCI has been found to increase the risk for subsequent cognitive decline [7]. Large-scale studies have found that up to 60% of patients with isolated SCI in the absence of traumatic brain injuries display various degrees of deficits in cognitive processing and emotional regulation [8–10]. Long-standing neuroinflammation due to pathological mechanisms activated by SCI are understood to be central to SCI-associated cognitive decline [11]. Cognitive dysfunction due to SCI can persist in the long term after discharge from hospital and adversely affect quality of life [12]. Differences between individuals in terms of post-SCI cognitive deficits are documented and attributed to multiple clinical factors including older age, history of smoking, lower educational attainment, premorbid cognitive ability, and more severe postconcussion symptoms [8, 12]. Such deficits are associated with SCI irrespective of the level of injury [9] and support the concept that neurological injury sustained during SCI causes remote pathological neurodegenerative changes in brain regions, as evidenced by longitudinal, long-term MRI changes noted in remote cerebral cortex and motor cortex regions [13–16]. Notably, the degree of adverse MRI changes at 6 months has been linked to worse long-term neuropathological and functional status [17].

Animal models of SCI show increased cell apoptosis, microglial activation, and neuronal cell loss in the hippocampus and cerebral cortex [18, 19]. SCI triggers widespread systemic inflammation with activation of multiple proinflammatory neuroimmune pathways [20]. A comprehensive understanding of detailed molecular mechanisms underpinning cognitive dysfunction after SCI is a subject of ongoing investigation. The course of SCI recovery is variable, and presently, functional or clinical evaluations are utilized for prognosis and clinical decision making [21]. However, improved and early identification of SCI patients at risk for secondary complications of neurological and cognitive dysfunction can enable timely neuroprotective and rehabilitative treatment to improve clinical outcomes. Currently, there is a need to identify clinically relevant biomarkers of cognitive impairment early during SCI [22].

Neuropeptide FF is an endogenous neuropeptide from the RFamide family with high levels in the mammalian CNS and is implicated in multiple physiological processes including pain-modulation by regulating opioid signaling, cardiovascular function, and neuroendocrine regulation including energy homeostasis [23–25]. Its actions are predominantly effected by NPFF1 and NPFF2 type receptors [26]. Neuropeptide FF and other RFamide peptides are shown to regulate hypothalamic pituitary axis activation [27]. Neuropeptide FF is secreted into the blood stream in a pulsatile manner [28] and into the cerebrospinal fluid [29]. Downstream effects of neuropeptide FF signaling involve the activation of nuclear factor kappa B (NF-*κ*B) pathway [30], which is centrally implicated in the pathophysiology of neuropathological and neurodegenerative disorders [31, 32]. Furthermore, the discovery of several neuropeptide FF ligands has demonstrated the potential for their application in multiple conditions [33]. SCI is known to induce the secretion of multiple neuropeptides and alter receptor levels as a part of the protective immunoinflammatory response [34] but the possible secretion of SCI induced neuropeptide FF into the bloodstream and its prognostic value for predicting subsequent cognitive impairment has not been investigated. The present prospective study is aimed at investigating serum neuropeptide FF levels after acute traumatic SCI as a predictor of subsequent cognitive decline.

## 2. Methods

### 2.1. Study Population

Patients with spinal cord injury (SCI) who attended the “Neck-Shoulder and Lumbocrural Pain Hospital,” Shandong First Medical University and Shandong Academy of Medical Sciences from July 2018 to June 2021 were enrolled in this study. Inclusion criteria were 18-80 years old with spinal fracture after trauma; hospitalization within 24 hours after injury, no history of previous neurological injury, brain injury, and no serious infectious diseases. Exclusion criteria were cardio-cerebrovascular, liver, or kidney diseases, or other serious systemic diseases, congenital spinal malformations, history of spinal surgery, patients with unstable vital signs, death with 14 days of injury, or unwillingness to participate. In addition, 60 age- and gender-matched healthy volunteers undergoing physical examination in the same hospital were recruited as healthy controls. All study procedures were compliant with the Declaration of Helsinki. The study protocol was approved by the ethics committee of the Shandong First Medical University and Shandong Academy of Medical Sciences. All patients or their legal guardians provided signed informed consent prior to recruitment.

### 2.2. Clinical and Laboratory Data

Clinical data including age, gender, and medical history (hypertension, diabetes, and atrial fibrillation) were collected. At the same time, the laboratory investigations were performed, including low-density lipoprotein, high-density lipoprotein, fasting blood glucose, total triiodothyronine (TT3), total thyroxine (TT4), and thyroid-stimulating hormone (TSH) levels. All clinical data were collected by the doctor in charge or nurses using questionnaires and patient records, and the laboratory data were recorded by a dedicated researcher.

### 2.3. Cognitive Function Test

All study participants were tested for cognitive function using Montreal cognitive assessment (MoCA) [35], 3 months after enrollment in the study. The MoCA is a widely applied cognitive screening tool, which has been translated into Chinese, validated, and widely applied [36]. The MoCA scale has a total score of 30 points. A higher score indicates a higher cognitive level. A threshold of 26 points has been commonly utilized for diagnosing cognitive dysfunction. The researcher recording the MoCA scores was blinded to subject grouping and baseline characteristics.

### 2.4. Serum Neuropeptide FF Levels

Fasting venous blood was collected from all study participants. The collection time was between 8 and 10 am on the morning following admission. Serum was obtained from venous blood by centrifugation, and enzyme-linked immunosorbent assay (ELISA) was performed immediately or after cryopreservation using commercially available reagents (MyBiosource, Inc., San Diego, CA, USA) with a detection range of 12.35-1,000 pg/mL.

### 2.5. Statistical Analysis

All statistical analyses were performed using SPSS 20.00 statistical software. Count data were expressed as percentages, and comparisons between groups were made using chi-square test. Continuous data (with normal distribution) were expressed as mean ± standard deviation and compared using student's *t*-test. SCI patients were further grouped based on quartile distribution of serum neuropeptide FF levels, and MoCA scores were compared between the 4 quartile groups using ANOVA test. Finally, multivariate linear regression analysis was performed for MoCA scores as outcome, with baseline clinical and laboratory characteristics as predictors in order to adjust for any additional risk factors of cognitive decline after SCI. *p* < 0.05 indicated a statistically significant difference.

## 3. Results

### 3.1. Baseline Clinical and Laboratory Characteristics

A total of 88 SCI patients and 60 control subjects were analyzed. As shown in Table 1, there were no significant differences between the two groups in age, gender, medical history, LDL, HDL, FBG, TT3, TT4, and TSH (*p* < 0.05).

**Figure 1:**
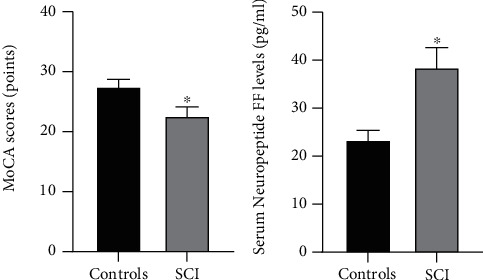
Differences in MoCA scores and serum neuropeptide FF levels between SCI patients and healthy controls. MoCA: Montreal cognitive test; SCI: spinal cord injury. Compared with the controls, ^∗^*p* < 0.05.

MoCA (controls (27.5 ± 1.2) points vs. SCI (22.6 ± 1.5) points) and serum neuropeptide FF levels (controls (23.4 ± 2.0) pg/m vs. SCI (38.5 ± 4.1) pg/m) were highly significantly different (*p* < 0.001) between SCI patients and healthy controls (Table 1 and Figure 1).

### 3.2. Relationship between MoCA and Serum Neuropeptide FF Levels in SCI

Table 2 presents the mean MoCA scores of SCI patients within each quartile of serum neuropeptide FF levels. A trend of decreasing MoCA scores with higher serum neuropeptide FF levels after SCI was noted, and the intergroup differences were highly significant (*p* < 0.001).

### 3.3. Multivariate Regression Analysis

Table 3 presents the outcome of multivariate regression analysis with the predictors, age, gender, LDL, HDL, FBG, TT3, TT4, and TSH levels and serum neuropeptide FF levels and showed that after adjusting for these baseline characteristics, neuropeptide FF levels showed significant predictive value for cognitive decline after SCI. Therefore, serum neuropeptide FF levels are an independent risk factor for cognitive impairment after SCI (*β* = 0331, *p* = 0.034).

## 4. Discussion

The present prospective cohort study demonstrated that serum neuropeptide FF levels measured on the day following hospital admission for SCI due to acute trauma could predict significant cognitive decline after 3 months. This relationship was evident after adjusting for clinical variables age, gender, and multiple laboratory metabolic variables including lipid levels, hyperglycemia, and thyroid hormones. Serum neuropeptide FF levels after SCI were found significantly elevated as compared to a group of healthy volunteers of comparable clinical and metabolic profiles. This finding suggests that neurological injury sustained during SCI initiates a rise in peripheral neuropeptide FF levels early during the postinjury period as part of the reparative immunoinflammatory response. Endogenous neuropeptides released after SCI are understood to mediate secondary injury in the brain, and the application of opioid antagonists has shown beneficial effects [37]. In addition, a neuropeptide FF-amide peptide precursor has been implicated in recovery from neurological injury in diabetes via the neuropeptide FF receptor 2 [38]. However, the effects of neuropeptide FF are understood to be pleiotropic, chiefly depending on receptor subtype and location. The neuropeptide FF receptors 1 and 2 have shown opposing effects in experimental data, whereby neuropeptide FF receptor 1 mediated pronociceptive effects whereas as neuropeptide FF receptor 2 attenuated proinflammatory and neuropathic pain [24]. Notably, about 5% of excitatory interneurons in the mouse spinal cord superficial dorsal horn region, responsible for the relay of noxious, thermal, or sensory stimuli, have been found to be neuropeptide FF expressing cells, forming a distinct cell population [39] but the spinal cord shows expression of neuropeptide FF receptor 2 alone whereas both 1 and 2 receptor subtypes are expressed in the brain, which begets the hypothesis that neuropeptide FF release after acute SCI may have differential effects in spinal cord and brain tissue owing to different receptor distribution patterns.

In the present study, high early neuropeptide FF levels were found to predict significantly worse cognitive function after 3 months among patients without any prior history of neurological dysfunction or concomitant traumatic brain injury. Traumatic brain injury or premorbid cognitive decline in acute SCI is associated with significantly worse neuropsychological functioning [8, 40] and can act as confounders and was therefore excluded. This relationship remained after controlling for several risk factors of cognitive decline. Age and gender are associated with differences in cognitive function [41]. Hyperglycemia has been linked to worse outcomes in SCI and cognitive function [42] and was therefore addressed as a possible covariate. Thyroid function has also been recognized as a moderator of cognitive dysfunction in middle- and older-aged populations by modulating attention processing and memory among other cognitive processes [43]. Similarly, very high or low LDL and low HDL levels have shown an association with cognitive decline among elderly individuals [44, 45], while chronic hypertension is a recognized as a risk factor for cognitive decline and dementia [46]. Furthermore, the regression analysis indicated none of these demographic and laboratory markers could significantly predict cognitive decline after SCI. Other possible contributors to secondary cognitive impairment after SCI include simultaneous occult brain injury, sleep apnea, and decentralized cardiovascular control [9]. Accurate prediction of post-SCI neurological function recovery from both primary and secondary injury remains a challenge [47]. Future studies must include population-scale data that combines multiple clinical, imaging, metabolic, biomarkers, and genomic data and leverages data science approaches to enable accurate prognostication of cognitive decline post-SCI.

The current study provides evidence for a plausible role of neuropeptide FF in mediating secondary cognitive dysfunction after SCI. The pathophysiology of neuropeptide FF in SCI-associated cognitive decline might be affected through multiple channels. SCI initiates a state of chronic neuroinflammation in the brain marked by increased levels of proinflammatory mediator chemokines and interleukin 6 [19, 48]. SCI also leads to increased endoplasmic reticulum stress, reduction in neurogenesis, and lower neuronal survival in several brain regions including the hippocampus, accompanied by immune activation of microglial cells [19, 49]. The role of neuropeptide FF signaling in these mechanisms is yet to be elucidated in detail. Neuropeptide FF signaling has been implicated in the potentiation of acute neuroinflammation and could be ameliorated by a selective neuropeptide FF antagonist [50]. Moreover, acute inflammation is shown to induce neuropeptide FF positive neurons in the spinal cord [51]. Notably, in an animal study, the activation of neuropeptide FF receptor 2 was demonstrated to potentiate inflammation-mediated depression [52], and thus a similar role may be hypothesized for cognitive dysfunction in post-SCI inflammation. SCI is followed by alterations in the hypothalamic-pituitary axis [53], which is critically involved in regulation of mental functioning and mood regulation [54]. As neuropeptide FF receptor signaling is a key pathway implicated in regulation of hypothalamic pituitary signaling [55], its activation may be implicated in post-SCI cognitive decline via the hypothalamic-pituitary axis dysfunction. In addition, neuropeptide FF is involved in the central regulation of blood pressure [25] and deregulation of centralized blood pressure control after SCI with adverse changes leading to hypertension are independently implicated as mechanisms promoting cognitive deficits after SCI [56]. Comprehensive investigation of potential multiple mechanisms of neuropeptide FF involvement in post-SCI cognitive decline is warranted, and the insights obtained may unravel potential applications of neuropeptide FF ligands in this context.

Here, serum was used for measuring circulatory neuropeptide FF levels. As neuropeptide FF levels in peripheral circulation are understood to arise chiefly due to leakage from CNS tissues [57] and peripheral tissues show limited expression [58], circulating neuropeptide FF level may be considered a surrogate of its CNS expression and thereby possess value as a biomarker. However, the nature of relationship between central and peripheral neuropeptide FF levels needs further confirmation. Similar to neuroendocrine hormones, a pulsatile pattern of secretion of neuropeptide FF in blood has been documented [27].

The linear negative relationship between circulating neuropeptide FF levels after SCI with the degree of post-SCI cognitive dysfunction provides strong basis for its clinical relevance as a biomarker. The present study did not assess the correlation of SCI severity with circulating neuropeptide FF levels, although these were found to correlate with the severity of post-SI cognitive dysfunction. Future studies should address possible association of circulating neuropeptide FF levels with the degree of SCI injury, as several biomarkers with linear association with SCI severity have been identified [59]. Individual differences in neuropeptide and other mediators release after neuronal injury may be attributed to multiple mechanisms. Genetic, epigenetic, and systemic factors may account for such intersubject variability in neuroimmune responses [60, 61]. Limitations of the current study include a single time-point sampling, limited sample size, lack of information on education level, and no dynamic detection of neuropeptide FF serum levels. Future large-scale studies are essential to validate the prognostic relevance of serum neuropeptide FF levels to predict the temporal course of cognitive decline after SCI. The present data also suggest a possibility for neuropeptide FF ligands to be applied for the management of post-SCI cognitive decline.

## 5. Conclusions

In sum, the present study demonstrated a significant prognostic value of serum neuropeptide FF levels to predict cognitive decline 3 months after SCI, after adjusting for multiple demographic and metabolic factors. Furthermore, higher post-SCI serum neuropeptide FF levels predicted worse cognitive status. These findings support serum neuropeptide FF levels as a biomarker and potential therapeutic target for improved management of cognitive dysfunction after SCI.

## Figures and Tables

**Table 1:** Characteristics of participants.

	Controls (*n* = 60)	SCI (*n* = 88)	*p* value
Age, years	57.8 ± 6.9	58.1 ± 7.2	0.801
Gender, male, *n* (%)	47 (78.3%)	64 (72.7%)	0.439
HP, *n* (%)	25 (41.7%)	36 (40.9)	0.927
DM, *n* (%)	18 (30%)	30 (34.1%)	0.602
AF, *n* (%)	5 (8.3%)	9 (15%)	0.699
LDL, mmol/L	2.5 ± 0.9	2.6 ± 0.9	0.508
HDL, mmol/L	1.2 ± 0.3	1.2 ± 0.2	1.000
FBG, mmol/L	6.3 ± 1.4	6.4 ± 1.6	0.695
TT3, *μ*g/L	1.2 ± 0.1	1.2 ± 0.2	1.000
TT4, *μ*g/L	80.1 ± 10.4	80.3 ± 10.7	0.910
TSH, uIU/mL	1.5 ± 0.8	1.6 ± 0.9	0.489
MoCA score	27.5 ± 1.2	22.6 ± 1.5	<0.001^∗∗^
Neuropeptide FF, pg/ml	23.4 ± 2.0	38.5 ± 4.1	<0.001^∗∗^

HP: hypertension; DM: diabetes; AF: atrial fibrillation; LDL: low-density lipoprotein; HDL: high-density lipoprotein; FBG: fasting blood glucose; TT3: total triiodothyronine; TT4: total thyroxine; TSH: thyroid-stimulating hormone; MoCA: Montreal cognitive test. ^∗^*p* < 0.05, ^∗∗^*p* < 0.001.

**Table 2:** Relationship between serum neuropeptide FF levels and cognitive function.

	Serum neuropeptide FF quartile				
	*Q*1 (*n* = 22)	*Q*2 (*n* = 22)	*Q*3 (*n* = 22)	*Q*4 (*n* = 22)	*p* value
MoCA score	25.3 ± 1.6	23.6 ± 1.4	22.3 ± 1.3	19.2 ± 1.7	<0.001^∗∗^

MoCA: Montreal cognitive test. ^∗^*p* < 0.05, ^∗∗^*p* < 0.001.

**Table 3:** Multivariate linear regression analysis to predict cognitive function in SCI patients.

	Regression coefficient	*p* value	95% CI
Age	0.305	0.477	0.213-1.264
Gender	0.249	0.126	0.143-1.308
LDL	0.184	0.245	0.099-1.051
HDL	0.216	0.301	0.174-1.285
FBG	0.097	0.213	0.031-1.376
TT3	0.143	0.469	0.118-1.279
TT4	0.128	0.278	0.075-1.193
TSH	0.152	0.412	0.082-1.213
Neuropeptide FF	0.331	0.034∗	0.236-0.897

HP: hypertension; DM: diabetes; AF: atrial fibrillation; LDL: low-density lipoprotein; HDL: high-density lipoprotein; FBG: fasting blood glucose; TT3: total triiodothyronine; TT4: total thyroxine; TSH: thyroid-stimulating hormone. ^∗^*p* < 0.05, ^∗∗^*p* < 0.001.

## Data Availability

The data presented in the study may be made available from the corresponding author upon reasonable request.
